# Efficacy of Rezafungin on *Candida albicans* Endophthalmitis in a Rabbit Model

**DOI:** 10.20411/pai.v10i2.873

**Published:** 2025-10-21

**Authors:** John Saghir, Janet Herrada, Lisa Long, Erin San Valentin, Thomas S. McCormick, Mahmoud Ghannoum

**Affiliations:** 1 Center for Medical Mycology and Integrated Microbiome Core, Department of Dermatology Case Western Reserve University, Cleveland, Ohio; 2 University Hospital Cleveland Medical Center, Cleveland, Ohio

**Keywords:** Rezafungin, *Candida albicans*, Fungal Endophthalmitis, Echinocandin, Ocular Fungal Infection, Invasive Candidiasis

## Abstract

**Background:**

Endophthalmitis, a severe infection of the intraocular tissues that can result in permanent loss of vision if not immediately treated, is often caused by fungi, namely *Candida albicans*. Treatment options are limited due to a lack of ocular penetration of antifungal drugs. Rezafungin, an echinocandin antifungal with a long half-life, which was recently approved by the US Food and Drug Administration (FDA), has shown efficacy against candidiasis.

**Methods:**

In this study, using a rabbit model, we compared rezafungin, micafungin, and voriconazole in a hematogenous *C. albicans* endophthalmitis rabbit model. Fungal burden was determined in the aqueous humor, vitreous humor, choroid-retina, and the kidneys of infected rabbits; eye lesions were visualized by indirect ophthalmoscopy.

**Results:**

No fungal growth was detected in the aqueous humor, vitreous humor, or choroid-retina of rabbits treated with 10 mg/kg rezafungin at the time of fungal inoculation. Additionally, rabbits given 10 mg/kg rezafungin showed the lowest kidney fungal burden (average log colony-forming units [CFUs]/g of < 0.5). In contrast, animals given either micafungin (6.2 mg/kg) or voriconazole (10 mg/kg) in the same treatment regimen were positive for fungal infection as measured by CFUs in each of these areas, demonstrating fungal burden. Additionally, significant increases in eye lesion scores were observed in rabbits given either micafungin or voriconazole, while no eye lesions were noted in rabbits that received rezafungin.

**Conclusion:**

Taken together, these results indicate that rezafungin was effective at reducing the acute fungal burden and subsequent eye lesions caused by *C. albicans*-induced endophthalmitis.

## INTRODUCTION

Endophthalmitis is a severe infection of the intraocular tissues that threatens both the aqueous and vitreous humor. This infection ultimately results in inflammation that can damage an individual’s vision, particularly in immunocompromised patients, resulting in permanent and devastating loss of vision if not treated withn days of initial infection [1–3]. The nidus of infection for endophthalmitis is typically exogenous infection, postoperative or post-trauma; however, endogenous (an infection caused by microbes already present in the body) endophthalmitis has also become increasingly relevant as it often occurs as a complication of disseminated candidiasis [4, 5].

While both bacterial and fungal organisms can be causative of endophthalmitis, *Candida* species are of particular relevance as they have been identified on contaminated medical tools in cases of exogenous infection, and are implicated in hematogenous spread in endogenous cases [6]. Fungal endophthalmitis is of specific clinical significance due to the potential for hematogenous spread, as well as the possibility of systemic spread, which may involve other organs in the process [7]. *C. albicans* accounts for 60% to 65% of fungal endophthalmitis cases [1], making this species of great importance in diagnosis and treatment of fungal endophthalmitis. Vitrectomy, a surgical procedure where the vitreous humor gel is removed, has been used as treatment to improve visual acuity; however, it is rather invasive [8]. Studies attempting to use corticosteroids to reduce inflammation *in vivo* have been unreliable [2]. More importantly, despite the availability of antifungal agents, effective treatment against endophthalmitis remains challenging due to limited ocular penetration of certain drugs.

Penetration issues have been highlighted in studies evaluating the efficacy of caspofungin, fluconazole, and voriconazole, all of which have failed to effectively treat fungal endophthalmitis caused by *Candida*, citing difficulty in their delivery to the eye [9–11]. Thus, there is an unmet need to successfully treat this devastating disease. Echinocandins, including caspofungin, micafungin, and anidulafungin, have been reported to exhibit poor penetration into ocular compartments due to their large molecular weight and high protein binding, rendering them suboptimal for treating fungal endophthalmitis despite being first-line therapy for candidemia [12, 13]. In contrast, azoles such as fluconazole and voriconazole achieve therapeutic levels in the aqueous and vitreous humor and are frequently used in the treatment of intraocular fungal infections [14].

Voriconazole in particular is noted for its broad-spectrum activity against *Candida* spp., including many non-*albicans* species and molds such as *Aspergillus* [15]. Flucytosine also achieves high intraocular concentrations due to its low molecular weight and minimal protein binding, making it a valuable adjunct in certain clinical scenarios [16].

Newly published 2025 European Confederation of Medical Mycology (ECMM), International Society for Human and Animal Mycology (ISHAM), and American Society for Microbiology (ASM) (ECMM–ISHAM–ASM) global guideline for invasive candidiasis [17], recommend azole agents—specifically fluconazole and voriconazole—as firstline systemic therapy for ocular *Candida* infections, including chorioretinitis and endophthalmitis, provided the isolate is susceptible. Liposomal amphotericin B (LAmB), at 3–5 mg/kg daily, is recommended as the primary alternative when azoles are contraindicated, ineffective, or in resistant strains, and when susceptibility is unknown. At present, echinocandins are not recommended for routine ocular candidiasis therapy, except potentially in early-stage chorioretinitis lacking vitreal involvement, where combination therapy may be considered as adjunctive treatment.

Rezafungin, a novel semisynthetic echinocandin with an extended half-life, has shown promise in treating invasive candidiasis with less frequent dosing compared to traditional antifungals [18]. Rezafungin was recently approved (2023) for use in adults with invasive *Candida* infection, in the EU, UK, and the United States [19]. As defined by cure rates and mortality, rezafungin has been shown to possess non-inferior efficacy to first-generation echinocandins such as caspofungin and is approved to be taken on a weekly rather than a daily basis, which increases the advantage of drug exposure. Previous studies of fungal endophthalmitis using rabbit models have investigated micafungin and caspofungin and have established starting points for effective concentrations [20]. However, the efficacy of rezafungin for fungal endophthalmitis has not yet been examined.

In this study, we evaluated rezafungin compared to voriconazole or micafungin in a rabbit endophthalmitis model of hematogenous *C. albicans* infection. The antifungal activity of rezafungin was assessed by measuring fungal burden in ocular tissues and evaluating clinical lesions and severity in the eyes. The effect of rezafungin on kidney tissue burden was also evaluated.

## MATERIALS AND METHODS

### Minimum Inhibitory Concentration (MIC) Testing

Initially, MIC of the infecting strain (*C. albicans* SC5314 isolated from a clinical invasive candidiasis sample) against rezafungin, micafungin, and voriconazole was conducted following the Clinical and Laboratory Standards Institute (CLSI) document M27-Ed4, 2017 [21]. The goal of this MIC test was to confirm whether the strain was susceptible to rezafungin and comparators (Table 1).

**Table 1.** Susceptibility Data for the Test Compounds Against the Infecting *C. albicans* SC5314 Strain (µg/mL)

Test Compound	C. albicans SC5314
Rezafungin	0.016
Micafungin	≤0.016
Voriconazole	0.008

### *In vivo* Antifungal Activity

Animal experiments were performed upon review and approval of a protocol by the Case Western Reserve University Institutional Animal Care and Use Committee (protocol number 2018-0083). All procedures in the protocol followed the Animal Welfare Act, the Guide for the Care and Use of Laboratory Animals, and the Office of Laboratory Animal Welfare.

Female New Zealand White rabbits were obtained from Covance, Inc., with a body weight of 2.5–3.5 kg. Animals were allowed to acclimate for a minimum of 7 days prior to conducting the experiment. Environmental controls for the animal room were set to maintain a temperature of 16–22 °C, a relative humidity of 30% to 70%, and a 12h:12h light-dark cycle.

Rabbits were pre-sedated with acepromazine and butorphanol and anesthetized with isoflurane prior to inoculation. Silicone catheters were placed into the jugular vein and tunneled subcutaneously as described previously [22]. Forty-eight hours after catheter implantation, the animals were injected intravenously (IV) with 5 × 10^6^ colony-forming units (CFUs) of *C. albicans* SC5314, resuspended in 2 mL of phosphate-buffered saline (PBS), via the catheter. Animals were divided into 4 groups: 1) rezafungin 10 mg/kg (200 mg human equivalent), 2) micafungin 6.2 mg/kg (100 mg human equivalent), 3) voriconazole 10 mg/kg (133 mg human equivalent), and 4) vehicle-treated control group. The test compounds were prepared in accordance with the manufacturer’s instructions. Specifically, VFEND Vials containing 200 mg lyophilized voriconazole were reconstituted with sterile water for injection to produce a solution containing 10 mg/mL. Based on the weight of the animals, the appropriate concentration of stock was further diluted in sterile water for injection to a final volume of 6 mL. Mycamine 50 (micafungin) mg vials were reconstituted with 5 ml of 0.9% saline and protected from light. Based on the weight of the animals, the appropriate concentration of stock was further diluted in 5% dextrose injection to a final volume of 6 mL. Rezafungin powder was reconstituted in a stock solution of 2 mL 0.9% saline for injection. Based on the weight of the animals, the appropriate concentration of stock was further diluted in 0.9% saline for injection to a final volume of 6 mL. All compounds were administered at 2 mL increments every 30 minutes for 2 hours via slow IV bolus via catheter at 0 and 80 hours post challenge; animals were followed for up to 8 days post inoculation. Each treatment group was allotted 6 animals. However, several animals were removed from the study due to catheter failure. Therefore, the rezafungin-, micafungin-, voriconazole-, and vehicle–treated groups had 6, 5, 4, and 5 rabbits, respectively.

### Clinical Lesion Severity Measurement

Immediately prior to euthanasia at day 8, the rabbits were anesthetized and their eyes examined for lesions. Both eyes were dilated with drops of 1% Atropine Sulfate Ophthalmic Solution (Akorn, Inc.) and 0.5% Tropicamide Ophthalmic Solution (Akorn, Inc.), and eye lesions were visualized by indirect ophthalmoscopy. Research staff were trained by third year ophthalmology resident Gustavo Munguba, MD, PhD, on proper methods for dilating and evaluating the fungal lesions in the eye. An ION Vision eZView 20D Lens was used during the examinations. Each eye was evaluated independently using a standard numerical score based on the number and size of the lesions as well as the total ocular damage caused by infection of the eye. The severity of each lesion was scored on a scale of 1+ to 4+, as follows: 1+, lesion barely visible; 2+, lesion small but easily visible; 3+, lesion large but less than 1 disk diameter in size; and 4+, lesion larger than 1 disk diameter. The total eye severity score was equal to the sum of the number of eye lesions multiplied by their severity scores [23]. The entire vitreous aqueous humor from each eye was collected, weighed, and homogenized.

### Fungal Burden Quantification in Tissues

The entire vitreous humor sample was plated onto Sabouraud dextrose agar (Hardy Diagnostics). Kidney and choroid-retina samples were also collected, weighed, homogenized, and serially diluted in normal saline (0.085%) for enumeration of tissue fungal burden, calculated based on average log CFUs/g for the tissue samples [24].

### Statistical Analysis

To compare values obtained from experiments related to fungal burden and clinical lesions, one-way ANOVA with a Bonferroni post-hoc test was employed in determining significance. All statistical analyses were performed using SPSS for Windows, version 29.0. A *P*-value of < 0.05 was considered statistically significant.

## RESULTS

### Fungal Burden Reduction in Tissues

**Figure 1.**
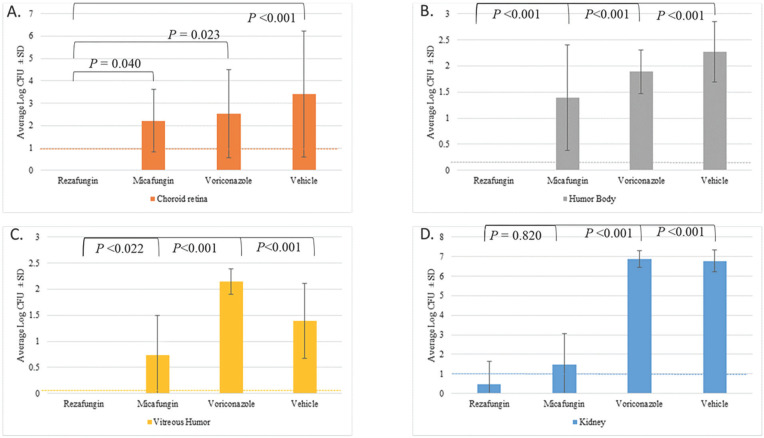
**Tissue fungal burden for the kidney, choroid retina, aqueous humor, and vitreous humor**^a^. ^a^Vitreous humor sample was collected in its entirety and plated directly. The limit of detection (LOD) for the ---- kidney and ---- choroid retina were both 10 CFUs, while the LOD for the ---- aqueous humor and ---- vitreous humor samples were 1 and 2 CFUs, respectively.

To evaluate the ability of the 3 antifungal treatments and vehicle-treated control to reduce fungal burden in ocular tissues, burden was assessed in key compartments of the eye. Tissue samples from the choroid retina, aqueous humor, vitreous humor, and kidneys were collected and analyzed for fungal burden. Figure 1 shows the average log CFUs/g ± SD for the tissue samples obtained.

In the choroid retina, the vehicle control group demonstrated the highest tissue fungal burden (average log CFUs/g ± SD of 3.42 ± 2.8). Animals given rezafungin had no detectable CFUs in either the right or left choroid retinas. In contrast, animals given micafungin or voriconazole showed fungal burdens of 2.21 ± 1.4 and 2.54 ± 2.0 log CFUs/g, respectively. Additionally, the rezafungin group showed significant antifungal activity when compared to the micafungin (*P* = 0.040), voriconazole (*P*=0.023), and vehicle control (*P*<0.001).

A similar trend was observed in the aqueous humor, where the vehicle control group exhibited the highest tissue fungal burden (2.27 ± 0.6 log CFUs/g). There were also no detectable CFUs in either the right or left humor bodies in animals given rezafungin. Animals given micafungin and voriconazole showed a fungal burden of 1.39 ± 1.0 and 1.89 ± 0.4 log CFUs/g, respectively. The rezafungin group showed significant antifungal activity when compared to the micafungin, voriconazole, and vehicle control groups (*P*<0.001).

The vehicle control group demonstrated a tissue fungal burden of 1.39 ± 0.7 CFUs/g ± SD in the vitreous humor. Similar to the other ocular compartments, animals given rezafungin had no detectable CFUs in either the right or left vitreous humors. In comparison, animals given micafungin and voriconazole showed a fungal burden of 0.74 ± 0.8 and 2.14 ± 0.2 log CFUs/g, respectively. The rezafungin group showed significant antifungal activity when compared to the micafungin (*P*=0.022), voriconazole (*P*<0.001), and vehicle control groups (*P*<0.001).

Fungal burden in the kidneys was evaluated to provide insight into the overall systemic efficacy of the antifungals. In the kidneys, the voriconazole and vehicle groups demonstrated the highest tissue fungal burden with 6.88 ± 0.4 log CFUs/g and 6.77 ± 0.6 log CFUs/g, respectively. Animals given rezafungin showed the lowest tissue fungal burden (0.47 ± 1.2 CFUs/g). The micafungin group had an average of 1.50 ± 1.4 log CFUs/g. The rezafungin and micafungingroups demonstrated a significant reduction in tissue fungal burden when compared to the vehicle control (*P*<0.001). There was no significant difference between the rezafungin and micafungin groups (*P*=0.820).

### Reduction in Clinical Lesion Severity

**Figure 2.**
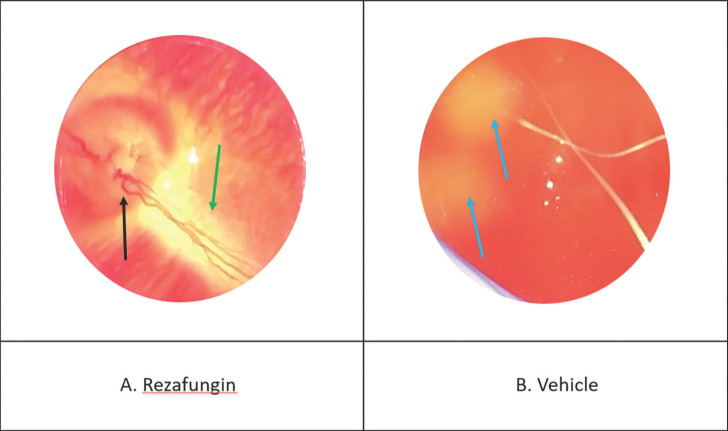
**Representative photographs of the indirect ophthalmoscopy**. Representative photographs of indirect ophthalmoscopy done for the (A) rezafungin and (B) vehicle-treated groups. As can be seen, the rezafungin–treated eye demonstrates a visible fundus (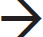), healthy optic disc (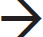), and blood vessels (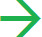) with no visible abnormalities. In contrast, the fundus of the vehicle–treated eye could not be visualized due to vitreal haze; additionally, there are 2 yellow-white fluffy lesions (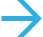) indicating *Candida* infection.

Table 2 shows the effects of treatment on the severity of *Candida* endophthalmitis as determined by indirect ophthalmoscopy scoring. Furthermore, Figure 2 shows representative photographs of indirect ophthalmoscopy done for: (A) rezafungin and (B) vehicle. As shown, the eye from animals administered rezafungin demonstrates a visible fundus (inner, back surface of the eyeball, opposite to the lens), healthy optic disc, and blood vessels with no visible abnormalities. In contrast, the fundus of the animals given vehicle alone could not be visualized due to vitreal haze; additionally, there are 2 yellow-white fluffy lesions indicating *Candida* infection. The vehicle control group demonstrated the highest eye severity scores with an average ± SD of 3.2 ± 2.5. No lesions were observed in the rezafungin group (average eye severity score of 0 ± 0). While animals given micafungin and voriconazole showed average eye severity scores ± SD of 1.9 ± 1.4 and 2.5 ± 1.8, respectively. The rezafungin group showed significant antifungal activity when compared to the voriconazole (*P*=0.007) and vehicle control groups (*P*<0.001). There was no significant difference between the rezafungin and micafungin groups.

**Table 2.** Effects of Treatments on the Severity of *Candida* endophthalmitis as Determined by Indirect Ophthalmoscopy Scoring

Treatment Group	Average Eye Severity Score ± SD
Rezafungin	0 ± 0^a,b^
Micafungin	1.9 ± 1.4
Voriconazole	2.5 ± 1.8
Vehicle Control	3.2 ± 2.5

a *P*<0.001 when compared to the vehicle control

b *P*=0.007 when compared to the voriconazole-treated group.

## DISCUSSION

Fungal endophthalmitis is challenging to diagnose and treat, as the infection can rapidly progress and lead to irreversible vision loss without effective intervention. Because intraocular *Candida* infections are often linked to systemic infections, systemic antifungal therapy is often reported, although agents need to cross the blood-retina barrier and achieve high ocular concentrations [25]. While intravitreal antifungal therapy such as voriconazole or amphotericin B can be effective, it often requires multiple injections and carries risks such as retinal toxicity [26]. Additionally, studies have attempted to address fungal endophthalmitis with drugs such as isavuconazole; however, these studies focused on *Aspergillus* species and require further investigation regarding their efficacy against candidal endophthalmitis [27]. Thus, there is a need to explore alternative strategies for addressing fungal endophthalmitis. In this study, we evaluated the antifungal activity of rezafungin on endophthalmitis caused by systemic *C. albicans* infection and quantified fungal burden in ocular tissues and kidneys, as well as assessing clinical lesion severity in a rabbit model, using voriconazole and micafungin as comparators.

The current data demonstrate that rezafungin was more effective than micafungin and voriconazole in reducing endophthalmitis in a rabbit model of *C. albicans* infection. As shown using eye fungal burden, as well as indirect ophthalmoscopy, rezafungin demonstrated significant antifungal activity when compared with animals administered either voriconazole or micafungin. No CFUs were detected in the aqueous humor, vitreous humor, or choroid-retina in the rabbits given rezafungin, and eye lesions were the lowest in the rezafungin groups, as there were no lesions observed. Since endophthalmitis associated with *Candida* infection is largely endogenous and thereby requires systemic therapy, it is important to note that rabbits in the rezafungin group demonstrated the lowest kidney fungal burden as well, demonstrating that this antifungal had broad target activity covering both eye infection as well as disseminated candidiasis.

The significant advantages that rezafungin demonstrates over its counterparts suggest that this therapeutic may have success in treating fungal endophthalmitis in other animal models, as well as more clinical success in patients. Not only does the reduction of fungal burden and prevention of ocular damage support this, but its increased half-life allows for prolonged sustainability of the drug level and gives rezafungin an advantage compared to other echinocandins. The increased half-life is attributed to the chemical structure of the compound, wherein a choline-derived moiety replaces the ornithine-derived hemiaminal structure seen in other echinocandins, leading to a more stable molecule [28]. The extended ability for rezafungin to be administered on a weekly basis rather than daily makes its therapeutic use far more practical [29].

Our study supports that rezafungin likely has sustained penetration into infected tissues. For example, in models of a liver abscess, rezafungin and micafungin have had their homogenous distribution compared, with rezafungin having more distribution in a shorter time frame, showing it can effectively distribute throughout infected tissue [30]. The overall pharmacokinetic profile of rezafungin includes its ability to inhibit the enzyme 1,3-β-D-glucan synthase, which helps to produce 1,3-β-D-glucan. This molecule is a crucial part of the cell wall of fungi, as without proper cell wall formation, there exists potential for osmotic instability and ultimately the death of the cell [26]. Micafungin functions similarly but lacks the structural stability and thus the extended half-life afforded by rezafungin [29]. Conversely, voriconazole is a triazole. Its mechanism involves interfering with ergosterol synthesis, a fungal membrane lipid [31]. This mechanism is less specific compared to rezafungin, and thus limits rather than stops cell growth of the fungi, as supported by the results of our study. Rezafungin is generally well tolerated in humans, with mild gastrointestinal and infusion-related reactions most frequently reported. Micafungin can rarely cause hepatotoxicity and hypersensitivity reactions. Voriconazole is associated with visual disturbances, hepatotoxicity, and phototoxicity

Future studies comparing the effect of rezafungin given twice a week versus voriconazole and micafungin given daily, are warranted as the difference in dosing between these agents is a variable in the current approach. In the current schema, rezafungin and the comparators were administered at the same time points (0- and 80-hours post inoculation), which favored rezafungin due to its longer half-life. However, future studies using rezafungin and comparators dosed daily are warranted, as well as comparative studies that use the approved daily dosing of micafungin and voriconazole, as well as additional comparators such as fluconazole, which achieves high concentrations in choroid/vitreous/retina [17]. Additionally, because this study was conducted in a rabbit model, additional studies validating the use of rezafungin in human clinical trials is necessary. Rezafungin can also be tested in combination with other antifungals to determine if a more effective treatment regimen is possible or whether antagonistic effects occur.

## CONCLUSION

This study showed that rezafungin possesses superior antifungal activity compared to voriconazole and micafungin in this rabbit model of *C. albicans* endophthalmitis, effectively reducing the fungal burden in ocular tissues and preventing lesion formation, as well as reducing kidney fungal burden. Given the challenges associated with current antifungal treatments, including the need for frequent dosing, limited ocular penetration, and potential toxicity, the extended dosing interval and enhanced tissue distribution observed for rezafungin make it a promising candidate for further clinical investigation, including studies designed to address treatment versus prophylaxis. Future studies should explore its efficacy in the clinical setting and evaluate its potential to reduce the need for invasive interventions such as vitrectomy and ultimately improve treatment outcomes for patients with fungal endophthalmitis.
